# Submicroscopic chromosome abnormalities associated with spina bifida (myelomeningocele)

**DOI:** 10.1186/1743-8454-7-S1-S20

**Published:** 2010-12-15

**Authors:** Dan Doherty, Karen D Tsuchiya, Heather C Mefford, David B Shurtleff

**Affiliations:** 1 Department of Pediatrics, University of Washington, Seattle, Washington, USA; 2 Department of Laboratories, Seattle Children's Hospital, Seattle, Washington, USA

## Background

Visible chromosome abnormalities have been commonly reported in patients with spina bifida (myelomeningocele), particularly when associated with other malformations. Newer technologies such as array comparative genomic hybridization (CGH) have made it possible to identify smaller (submicroscopic) DNA deletions/duplications, also referred to as copy number variants (CNVs). Submicroscopic CNVs have been identified as the cause of a variety of disorders, substantially increasing the diagnostic yield in patients with intellectual disability and multiple congenital malformations. Submicroscopic CNVs have not been commonly reported in patients with spina bifida, but given the high frequency of visible chromosome rearrangements, we hypothesize that submicroscopic CNVs also contribute to the genetic etiology of spina bifida.

## Materials and methods

We reviewed all array CGH results at a single children’s hospital laboratory for which neural tube defect was included in the indication for the test. During 2007-2009, array CGH was performed by the laboratory on a total of 1988 patients.

## Results

Fourteen patients with spina bifida were evaluated by array CGH during the study period. CNVs were identified in two patients: 1) A 14 year old male with thoracosacral myelomeningocele, diastematomyelia, shunted hydrocephalus, bilateral clubfeet, bilateral subependymal heterotopias, bilateral closed-lip schizencephaly, seizures and polyarticular juvenile rheumatoid arthritis, had normal high-resolution karyotype (550-650 band), but array CGH revealed a 1.3Mb interstitial deletion in16p13.11. Of note, the patient’s brother has Gorlin syndrome, but no neural tube defect, and carries the same deletion. Parents have not been tested. 16p13.11 deletion has been reported in patients with autism, intellectual disability, epilepsy, holoprosencephaly and other malformations, but also in a few phenotypically normal individuals. 2) A 14 year old female with thoracic myelomeningocele, shunted hydrocephalus, bilateral hearing loss and caudal regression with imperforate anus had a normal high-resolution karyotype (600-650 band), but array CGH revealed a 0.4-2.1 Mb duplication in 15q13.3. Mother does not carry the duplication, father has not been tested and a 17 year old sister with learning disabilities and ADHD has not been tested. 15q13.3 duplication has been reported in patients with intellectual disability, autistic behaviors and dysmorphic features.

## Conclusions

Newer technologies for detecting smaller chromosome abnormalities may identify the cause of spina bifida in a proportion of patients. We hypothesize that atypical presentation may be associated with increased risk of an underlying CNV. Larger studies are required to determine the prevalence of CNVs in patients with spina bifida and whether patients with CNVs display atypical clinical features.

